# Cost-Effectiveness Analyses of Home Parenteral Nutrition for Incurable Gastrointestinal Cancer Patients

**DOI:** 10.3389/fonc.2022.858712

**Published:** 2022-05-18

**Authors:** Wenqian Li, Hanfei Guo, Lingyu Li, Jiuwei Cui

**Affiliations:** Cancer Center, The First Hospital of Jilin University, Changchun, China

**Keywords:** home parenteral nutrition, cost-effectiveness analysis, gastrointestinal cancer, quality-adjusted life year (QALY), willingness-to-pay (WTP)

## Abstract

**Background:**

Appropriate nutritional support, including supplemental home parenteral nutrition (sHPN), may improve prognosis and quality of life (Qol) of malnourished cancer patients. We aimed to explore the cost-effectiveness of sHPN for incurable gastrointestinal cancer patients from the Chinese healthcare perspective.

**Method:**

Clinical data were extracted from a randomized controlled trial (NCT02066363). Patients were randomized into the sHPN group or the non-sHPN group (receiving best practice nutritional care). A Markov model was established with a 6-week cycle length. Costs were acquired from local hospitals, effect parameters included quality-adjusted life year (QALY), Qol, body mass index, fat-free mass (FFM), FFM index, handgrip strength, and a 6-min walking test. Sensitivity analyses were conducted with a willingness-to-pay (WTP) set at 3 per capita gross domestic product ($29,307/QALY).

**Results:**

When considering QALY as a utility, the incremental cost-effectiveness ratio (ICER) was $24,289.17, with an incremental cost of $2,051.18 and an incremental QALY of 0.0844 between the sHPN group and the non-sHPN group. Furthermore, we explored the cost-effectiveness of sHPN from multidimensions, where we analyzed various effect parameters at different visits; the results showed a superior benefit for patients in the sHPN group except for the handgrip parameter at visit 2. Sensitivity analysis demonstrated the influence of utilities in the sHPN group, but the sHPN group was still cost-effective with a WTP of $2,500/QALY.

**Conclusion:**

In China, sHPN was cost-effective for patients with incurable gastrointestinal cancer, which suggested further applications in clinical practice and provided references for clinical decisions and pricing.

## Introduction

Malnutrition is defined as an imbalance in nutritional compositions with negative effects on body functions and clinical outcomes in cancer patients, and it accounts for 20%–30% of deaths in terminal cancer patients (1–3). Over 80% of gastrointestinal cancer patients develop malnutrition (4). On the one hand, gastrointestinal cancers often cause digestive tract dysfunction and obstruction, which lead to insufficient nutrition and energy intake (5). On the other hand, antitumor therapy is likewise a factor of malnutrition that increases nutritional needs and develops inevitable side effects, including gastrointestinal symptoms that would further aggravate the nutritional problems of cancer patients (6).

Currently, the European Society for Parenteral and Enteral Nutrition (ESPEN) recommends that home parenteral nutrition (HPN) be administered to adult cancer patients who are unable to achieve nutritional requirements through oral food intake or enteral nutrition (EN) and are at risk of malnutrition death (7, 8). Patients with incurable gastrointestinal tumors may struggle to meet nutritional needs, whereas the use of supplemental HPN (sHPN) for heterogeneous cancer patients may result in a significant improvement in their quality of life, particularly in terms of physical and functional well-being, and even prolonged survival with manageable complications (5, 9–12). Studies also showed that appropriate nutrition support was related to better antitumor therapy tolerance and a reduction of therapy toxicity (13).

Nevertheless, the implementation of sHPN is still controversial. Despite the insufficient awareness and prompt treatment in daily clinical practice, some researchers believed that it is an expenditure of social resources with limited benefits (14, 15). In addition, it could increase the risk of infection, family burden, and generate additional costs, including costs of drugs, injections, training, complications, and readministrations (16). A recent randomized clinical trial (RCT) (NCT02066363) demonstrated the efficacy of sHPN in patients with incurable gastrointestinal cancers, suggesting an improvement in muscle condition [fat-free mass (FFM), fat-free mass index (FFMI)] and quality of life. However, it was a unilateral analysis without a consideration of social resources (17). Hence, we aimed to conduct a comprehensive analysis of the clinical effects and economic benefits of sHPN for incurable gastrointestinal cancer patients from the perspective of the Chinese healthcare system, with the goal of providing nutritional guidance to patients with advanced cancer.

## Method

### Patients and Clinical Data

The clinical data were from the open-label RCT, NCT02066363 (17). Patients were included if they were histologically diagnosed with locally advanced or metastatic gastrointestinal cancer, with a nutritional risk screening (NRS 2002) score of ≧2 representing nutritionally at risk. Other eligibilities contained age >18 years and performance status (PS) 0–2. Patients administered chemotherapy were not excluded. However, functional or actual short bowel syndrome was an exclusion criterion. A total of 234 patients were eligible for inclusion, with 47 patients accepted enrolment. A restricted randomization method minimization procedure was conducted between two groups: patients receiving sHPN and dietetic counseling in the sHPN group and patients receiving best-practice nutritional care and dietetic counseling in the non-sHPN group. Baseline characteristics of patients between the two groups were balanced and comparable in terms of cancer diagnoses, age, PS, treatments, and nutritional parameters. For patients in the sHPN group, patients were administrated with sHPN at 25%–35% of their daily nutritional needs, which was set as energy 125 kJ/kg, protein 1.5 g/kg/day, and fluid 35 ml/kg/day. Patients received sHPN for 2–4 days/week during nighttime. In the non-sHPN group, EN was allowed for patients who failed to meet nutritional requirements from general food intake. Treatments lasted for 24 weeks, with visits every 6 weeks by a dietician and the investigator. Homecare nurses were responsible for the administration of sHPN, and medications were prescribed by oncologists.

A Markov model was built by Treeage Software. It contained two Markov status: survive and die. The Markov cycle length was set as 6 weeks. Survival data were extracted from survival curves with Getdata Graph Digitizer Software (median overall survival (OS): 168 vs.169 days). We then simulated the curve with the Weibull model and used R software for the calculation of transformation probabilities (Pt) at cycle t using the following formula: Pt = 1–Exp[λ(t − u)^γ^–λt^γ^], where u, λ and γ represent the Markov cycle length, scale parameter, and shape parameter, respectively. Parameters are displayed in **Table 1**.

**Table 1:** Parameters of cost-effectiveness analyses.

Parameters	sHPN		Non-sHPN	
**Costs ($)**				
PN	421.58	Cycle	–	
CVC	64.48	Once	–	
CVC care	221.79	Cycle	–	
PICC	359.38	Once	–	
Homecare nurse	41.07	Cycle	–	
Ward	8.56	Once	–	
Readmission	365.70	Once	365.70	Once
EN	–		3.54	Once
Supportive care	675.00	Cycle	675.00	Cycle
Follow-up	111.20	Cycle	111.20	Cycle
**Utility**				
Baseline	0.60		0.64	
Visit 2	0.67		0.65	
Visit 3	0.69		0.53	
Visit 4	0.78		0.60	
Visit 5	0.69		0.56	
**Transformation parameters**				
Shape	0.89735193		0.95341849	
Scale	0.05464799		0.04435547	

sHPN, supplemental home parenteral nutrition; PN, parenteral nutrition; CVC, central venous access; PICC, peripherally inserted central catheter; EN, enteral nutrition.

### Cost

Costs were calculated based on the application of drugs and the report of hospitalizations in the NCT02066363 trial. Unit costs were acquired from local and community hospitals in China, including costs of parenteral nutrition (PN), EN, central venous access (CVC), peripherally inserted central catheter (PICC), and wages for homecare nurses. We assumed a typical patient with a height of 1.64 m, a weight of 65 kg, and a body surface area (BSA) of 1.72 m^2^ for the calculation of cost parameters. Costs of re-administration for patients receiving PN, as well as costs of follow-up and supportive care for patients with advanced cancer were obtained from other cost-effectiveness studies (18, 19). Since we focused on incremental benefits, we did not account for balanced cost items between the two groups, such as costs of dietetic counseling and chemotherapy (91% of patients received chemotherapy in the sHPN group, 92% in the non-sHPN group). Costs were measured in dollars with an exchange rate of 7.012; the discount rate was set at 3%. Cost parameters are displayed in **Table 1**.

### Effect

We extracted various effect parameters from the NCT02066363 trial, which explored the benefits of both long-term survival and short-term objective indicators, including quality of life, body mass index (BMI), FFM, FFMI, handgrip strength, and 6-min walking test (6MWT) at different visits. BMI was calculated by weight and height (BMI = weight/height^2^, kg/m^2^), while FFM was evaluated by bioelectrical impedance (BIA), and FFMI was a normalized index for FFM (FFMI = FFM/height^2^, kg/m^2^). Handgrip strength was estimated by a hand dynamometer according to the highest value after measuring three times, while 6MWT was performed on a marked 30 m walking distance. While effect parameters including FFM, FFMI, handgrip strength, and 6MWT were reflections of muscle function and performance, BMI represents basic body fat status, quality of life reflects the physical and emotional function of patients. We first explored quality-adjusted life year (QALY) considering both survival period and quality of life, and then we further evaluated various short-term effect indicators. Utilities are shown in **Table 1**.

### Outcomes

We conducted cost-effectiveness analyses in two steps from the Chinese healthcare perspective over a 5-year horizon. In the primary analysis, the incremental cost-effectiveness ratio (ICER) was evaluated, which estimated the benefit of costs and QALYs for the administration of sHPN. The secondary outcomes included various effect parameters, including quality of life, BMI, FFM, FFMI, handgrip strength, and 6MWT. The ICERs at different visits were compared as well. The willingness-to-pay (WTP) threshold was set at 3 per capita gross domestic product ($29,307/QALY).

Sensitivity analyses were carried out for the reliability of primary outcomes, including one-way sensitivity analysis and probabilistic sensitivity analysis. We assumed a 50% fluctuation in cost parameters, a 30% fluctuation both in transition probability parameters and utility values based on the data in the trial. The ranges of parameters are shown in **Supplementary Table S1**. One-way sensitivity analysis would be displayed in tornado diagrams. Probabilistic sensitivity analysis was conducted by a Monte Carlo simulation with 1,000 iterations. Utilities and transition probabilities were assumed to conform to the beta distribution while costs to fit gamma distribution (18). The results would be shown in cost-effectiveness acceptability curves and incremental cost-effectiveness scatter plots.

## Result

**Figure 1:**
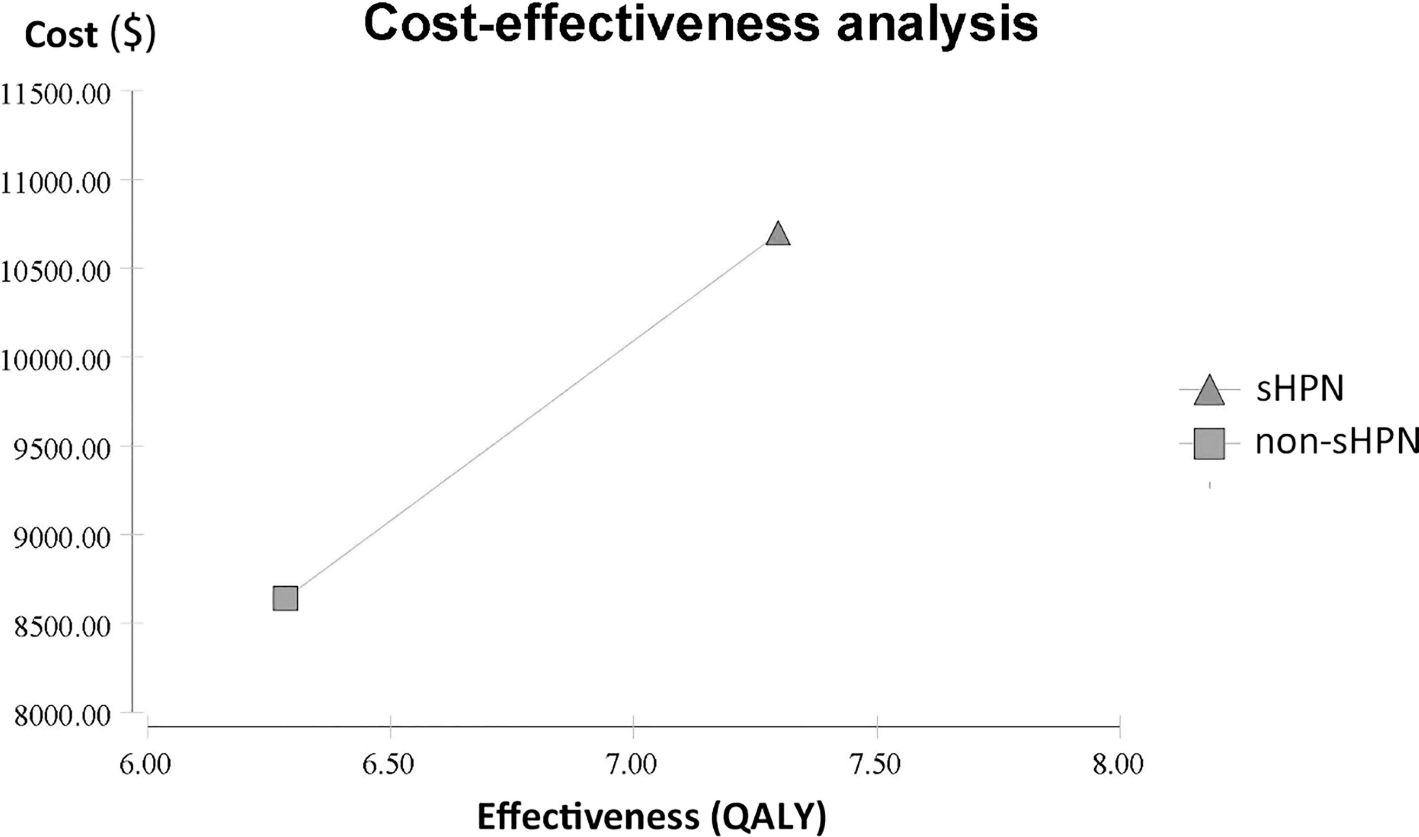
Cost-effectiveness analysis between the sHPN group and the non-sHPN group. QALY, quality-adjusted life year; sHPN, supplemental home parenteral nutrition.

For the primary outcome, cost-effectiveness analysis showed patients in the sHPN group spent $10,693.40, with a QALY of 0.6081, while patients in non-sHPN group spent $8,642.22, with a QALY of 0.5237. The incremental cost between the sHPN group and the non-sHPN group was $2,051.18, and the incremental QALY was 0.0844. The ICER was $24,289.17, which achieved an economic benefit for patients with HPN comparing with the WTP ($29,307/QALY) threshold in China. Results are shown in **Figure 1** and **Table 2**.

**Table 2:** Primary outcomes of cost-effectiveness analysis.

Group	E (QALY)	C ($)	IE (QALY)	IC ($)	ICER (QALY)
sHPN	0.6081	10,693.40	0.0844	2,051.18	24,289.17
Non-sHPN	0.5237	8,642.22	–	–	–

sHPN, supplemental home parenteral nutrition; E, effect; C, cost; IE, incremental effect; IC, incremental cost; ICER, incremental cost-effectiveness ratio; QALY, quality-adjusted life year.

For the secondary outcomes, we explored the difference from the baseline of each effect parameter and further calculated the corresponding ICERs between the sHPN group and the non-sHPN group at each visit. Results showed that outcomes in the sHPN group were superior to those in the non-sHPN group except for the measurement of handgrip at the time of visit 2. At the time of visit 3, three analyses achieved the best economic results, including quality of life, BMI, and FFM parameters, with an ICER of $9,004.58, $600.31/kg h^−2^, and $178.31/kg, respectively. Two analyses balancing FFMI and 6MWT parameters demonstrated the best economic benefits at the time of visit 2 with an ICER of $797.45/kg h^−2^ and $13.62/m, respectively. When considering handgrip as the effect parameter, the most cost-effectiveness result was achieved at visit 5, with an ICER of $411.67/kg. Results are listed in **Table 3** and **Supplementary Table S2**.

**Table 3:** Secondary outcomes of cost-effectiveness analyses.

ICER	Qol	BMI (kg h^−2^)	FFM (kg)	FFMI (kg h^−2^)	Handgrip (kg)	6MWT (m)
Visit 2	18,607.17	1,116.43	218.91	797.45	−1,014.94	13.62
Visit 3	9,004.58	600.31	178.31	857.58	2,001.02	20.24
Visit 4	11,297.28	4,142.34	443.82	1,462.00	2,259.46	16.24
Visit 5	18,646.40	7,924.72	495.29	2,438.37	411.67	26.42

BMI, body mass index; FFM, fat-free mass; FFMI, fat-free mass index; h, height; ICER, incremental cost-effectiveness ratio; kg, kilogram; m, meter; Qol, quality of life; 6MWT, 6-min walking test.

**Figure 2:**
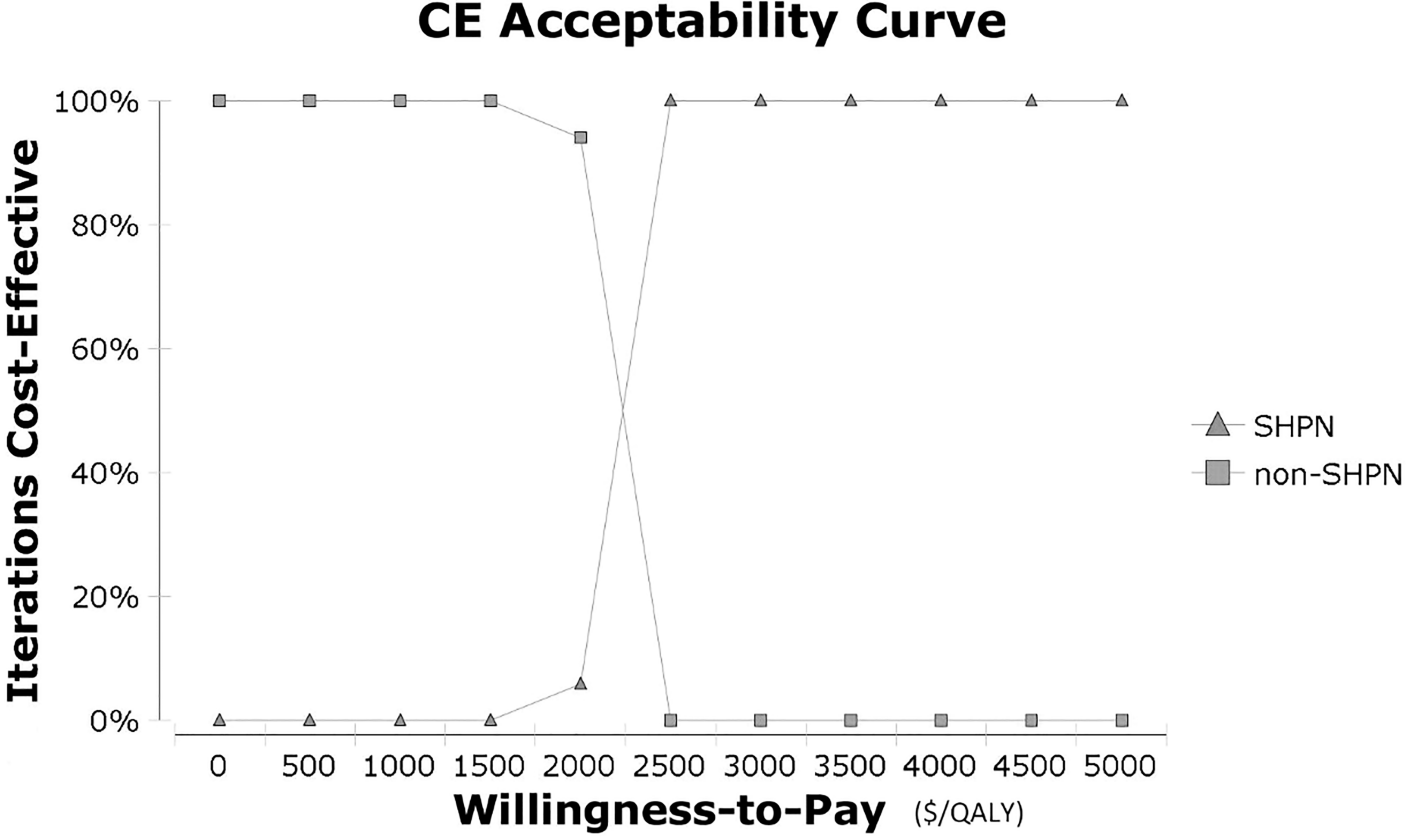
Cost-effectiveness acceptability curve in probabilistic sensitivity analysis. CE, cost-effectiveness; QALY, quality-adjusted life year; sHPN, supplemental home parenteral nutrition.

In the one-way sensitivity analysis, results showed the most influential parameter was the utilities in the sHPN group, followed by transformation probabilities in the sHPN group (**Supplementary Figure S1**). In the probabilistic sensitivity analysis, the cost-effectiveness acceptability curve (**Figure 2**) showed the sHPN group achieved economic benefits even if the WTP was set at $2,500/QALY. The incremental cost-effectiveness scatter plot (**Supplementary Figure S2**) showed that a Monte Carlo simulation with 1,000 iterations was stable.

## Discussion

The prevalence of HPN was reported to be approximately 5–79 per million inhabitants per year in western countries, and 25% of patients suffered from malignant diseases (20–22). However, HPN is not a routinely recommended method of nutritional support in China considering its actual burdens despite China’s high incidence of gastrointestinal cancers, especially gastric cancer and liver cancer (23). Thus, we explored the economic benefits of sHPN for incurable gastrointestinal cancer patients considering various effect parameters in the perspective of the Chinese health system. Through the combined analyses of clinical efficacy and economics, we hope to provide references for clinical applications and designations of Chinese national medical insurance policies, and also provide references for the pricing of their related costs in China.

In the current study, we conducted a cost-effectiveness analysis where we comprehensively analyzed cost, quality of life, and survival of incurable gastrointestinal cancer patients as the primary outcome. The ICER was $24,289.17, which showed the economic benefits of sHPN. Subsequently, we performed cost-effectiveness analyses based on the effect parameters provided in the clinical trial (17). Results showed that the economic benefits could be achieved on various clinical effects except for the handgrip parameter at the time of visit 2, and the period for the obtainment of the best economic benefits differs among effect parameters. It is noticeable that although we analyzed the differences between each parameter and their baseline values, it was improper for the comparison among parameters due to different units of effect parameters and ICERs. In summary, while obtaining clinical benefits, sHPN also achieved economic benefits and could be a recommended form of nutrition support for patients with incurable gastrointestinal tumors in China.

Previous economic analysis focused on patients with malignant inoperable bowel obstruction (IBO), which demonstrated that HPN was associated with poor economic benefits. The administration of HPN for patients with malignant intestinal failure should be under a comprehensive and careful consideration (24). Different inclusion criteria of patients might account for the result. In our analysis, HPN for incurable gastrointestinal cancer patients was used as a supplemental nutrition support, while for patients with malignant intestinal failure, parenteral nutrition support was the primary source of energy and nutrition. Thus, application dosage, complication rates, and corresponding costs all increased. It suggested that early supplemental nutrition support for advanced cancer patients with malnutrition could improve economic benefits and further confirmed the significance and feasibility of sHPN. Besides, in our study, sHPN was administrated at 25%–35% of the daily nutritional need for 2–4 days/week based on the NCT02066363 trial. Due to a lack of data, we did not explore the cost-effectiveness of different sHPN dosages or frequencies. Therfore, the best mode of sHPN administration still needs further exploration.

Since sHPN has not been extensively promoted in China yet, our research could be a reference for pricing. Costs were calculated according to current prices in China. We did not reckon on the cost of training for sHPN because free seminars and training were held regularly in Chinese hospitals for patients by doctors, nurses, and pharmacists. However, common complications of sHPN mainly include infections, catheter obstructions, and thrombosis, which raise the significance of catheter care training for patients and homecare nurses (12, 25). Therefore, when sHPN is widely accepted and applied in China, full-time personnel should be set up to take charge of training and be paid a corresponding fee. With a WTP of $29,307/QALY in China, economic benefits could still be achieved if the training fee was set as $423.75. Besides, if sHPN could be covered by Chinese national medical insurance, further cost reductions could be achieved by bearing the costs of more additional services such as dietitian, pharmacist, and other healthcare workers.

Our research has the following advantages. First, our study is the first cost-effectiveness analysis of sHPN for incurable gastrointestinal cancer patients. Previous economic analyses of nutrition support mainly focused on the effect indexes such as changes in hospitalization stays, complication rates, and related markers (26). Our study conducted cost-effectiveness analyses from multiple dimensions by evaluating sHPN on both long-term survival and short-term objective indicators, which provided nutritional options for clinical practice in China. In addition, our study focused on patients with incurable gastrointestinal cancers. Previous studies have shown that tumor type had little impact on the prognosis of incurable cancer patients receiving HPN, thus our economic analysis might also be suggestive for patients with other types of cancer (27, 28).

There are also several limitations. Firstly, despite the benefits of sHPN, there is still a lack of universally accepted standards for the application of sHPN. The current study was based on the NCT02066363 trial, and thus the indication of sHPN was consistent with the trial. However, considering the huge population base and rare medical resources in China, the specific indications and criteria of the sHPN application still need further exploration. Secondly, the current economic analysis was based on the Chinese healthcare perspective, since costs vary widely among regions, and thus our results were applicable to the Chinese healthcare system and were limited to other countries with different healthcare systems (7, 27). However, due to the lack of clinical data for Chinese patients, our analysis was based on a Danish unicentral open-label RCT, and thus ethnic heterogeneity should be taken into consideration as well. Despite the clinical characteristics and survival of patients in the NCT02066363 trial were comparable with those of Asian patients, differences in BMI existed among different populations. Patients in Europe had a slightly higher average value of BMI than Asian patients (25–26 vs. 23–24) (29–31). Besides, considering the limited sample size, clinical results should be further confirmed by large-scale clinical trials. The stability of the results should be considered, especially for 6MWT due to missing values (17). Thirdly, the accurate cost of best practice nutritional care for patients in the non-sHPN group was unavailable, while only the cost of EN was reckoned in. Finally, the impact of HPN on distinct individuals varies, depending on their underlying disease and performance state (32). Thus, individualized treatment should be applied to the administration of HPN.

In conclusion, malnutrition is a common comorbidity for patients with incurable gastrointestinal cancers. Considering the unmet nutritional needs, we conducted cost-effectiveness analyses on the application of sHPN for patients with incurable gastrointestinal cancer, which showed sHPN was cost-effective and worthy of further promotion in China.

## Data Availability Statement

The original contributions presented in the study are included in the article/**Supplementary Material**. Further inquiries can be directed to the corresponding author.

## Author Contributions

The manuscript has been read and approved by all the authors, that all journal requirements for authorship have been met, and that each author believes that the manuscript represents honest work.

## Funding

This work was supported by the National Natural Science Foundation of China [grant number 81874052]; Jilin Province Department of Finance [grant number JLSWSRCZX2020-0023]; Technology Development Program of Jilin Province [grant number 20190303146SF].

## Conflict of Interest

The authors declare that the research was conducted in the absence of any commercial or financial relationships that could be construed as a potential conflict of interest.

## Publisher’s Note

All claims expressed in this article are solely those of the authors and do not necessarily represent those of their affiliated organizations, or those of the publisher, the editors and the reviewers. Any product that may be evaluated in this article, or claim that may be made by its manufacturer, is not guaranteed or endorsed by the publisher.
