# Supplementary material for: Cost-Effectiveness Analyses of Home Parenteral Nutrition for Incurable Gastrointestinal Cancer Patients
Source: Front Oncol. 2022 May 18;12:858712. doi: 10.3389/fonc.2022.858712 (PMC9157576; doi:10.3389/fonc.2022.858712)
Supplement: Supplementary file 1 [file DataSheet_1.docx]

**Supplementary Fig 1.** Tornado Diagrams in one-way sensitivity analysis.


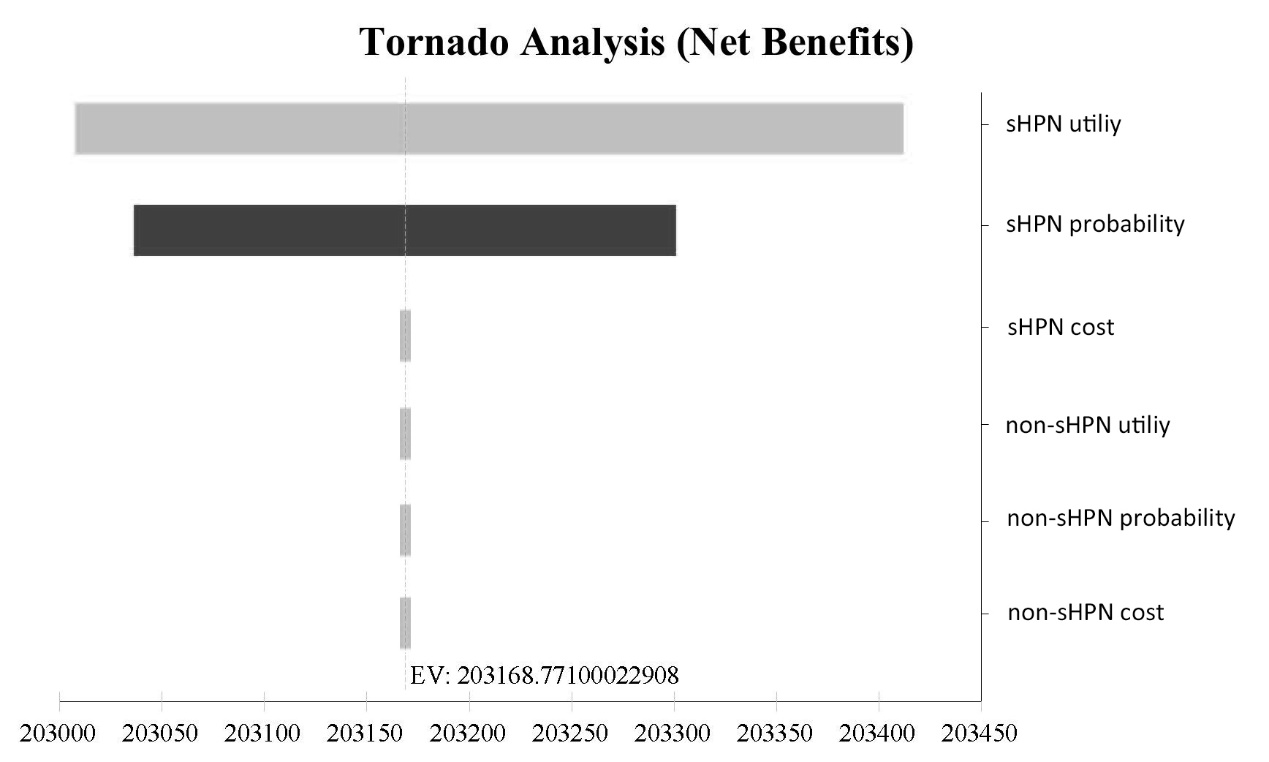


**Supplementary Fig 2.** Incremental cost-effectiveness scatter plots in probabilistic sensitivity analysis


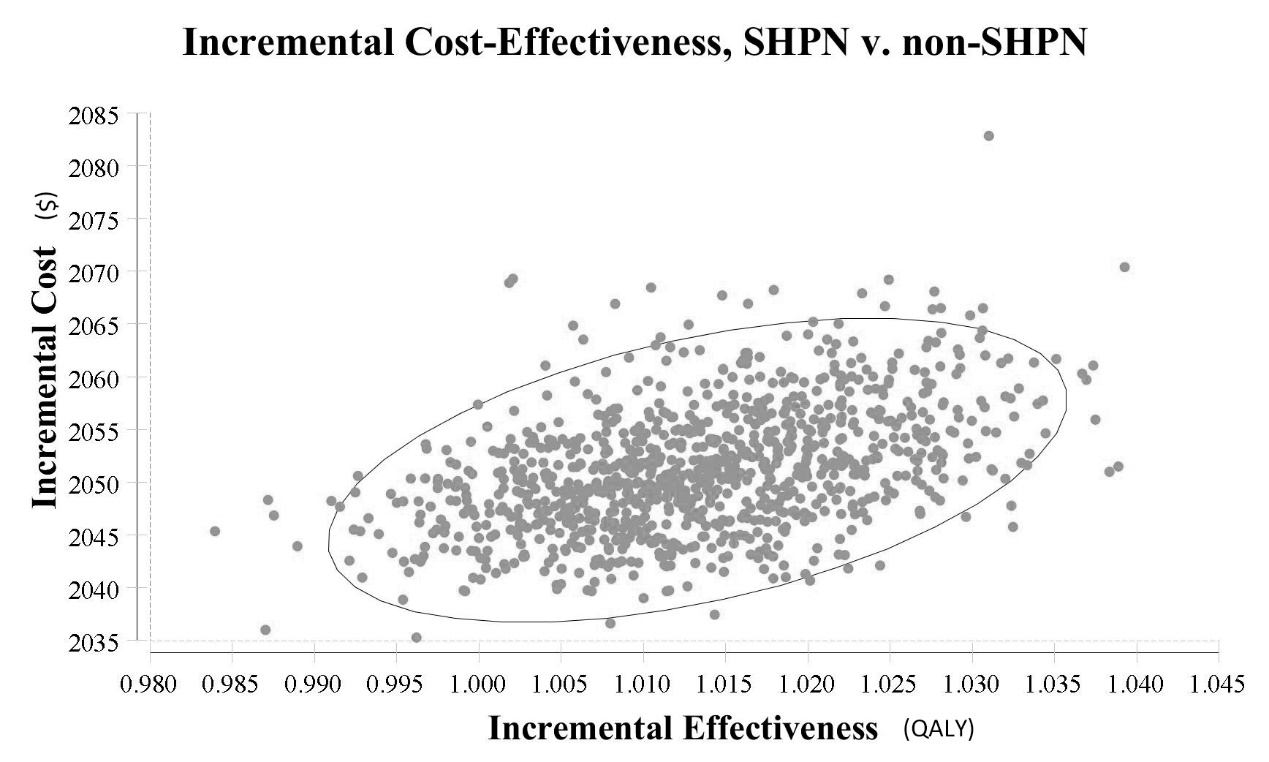


| **Supplementary table 1.** Ranges of parameters in sensitivity analyses | | | | |
| --- | --- | --- | --- | --- |
| group | parameter | basic level | low | high |
| sHPN | cost ($) | 2268.86 | 1134.43 | 3403.29 |
|  | utility | 0.64 | 0.60 | 0.78 |
|  | probability | 0.08 | 0.05 | 0.10 |
| non-sHPN | cost ($) | 1152.43 | 576.21 | 1728.64 |
|  | utility | 0.65 | 0.53 | 0.65 |
|  | probability | 0.06 | 0.04 | 0.08 |
| sHPN: supplemental home parenteral nutrition | | |  |  |

| **Supplementary table 2.** Results and detailed information of cost-effectiveness analyses | | | | | |
| --- | --- | --- | --- | --- | --- |
| Parameters | Visit | sHPN | non-sHPN | difference | ICER |
| cost ($) | Visit 2 | 2268.86 | 1152.43 | 1116.43 | - |
|  | Visit 3 | 3739.54 | 1938.63 | 1800.92 | - |
|  | Visit 4 | 5210.23 | 2724.83 | 2485.40 | - |
|  | Visit 5 | 6680.91 | 3511.03 | 3169.89 | - |
| Qol | Visit 2 | 0.07 | 0.01 | 0.06 | 18607.17 |
|  | Visit 3 | 0.09 | -0.11 | 0.20 | 9004.58 |
|  | Visit 4 | 0.18 | -0.04 | 0.22 | 11297.28 |
|  | Visit 5 | 0.09 | -0.08 | 0.17 | 18646.40 |
| BMI (kg^.^h^-2^) | Visit 2 | 0.70 | -0.30 | 1.00 | 1116.43 |
|  | Visit 3 | 1.20 | -1.80 | 3.00 | 600.31 |
|  | Visit 4 | 2.20 | 1.60 | 0.60 | 4142.34 |
|  | Visit 5 | 2.00 | 1.60 | 0.40 | 7924.72 |
| FFM (kg) | Visit 2 | 2.30 | -2.80 | 5.10 | 218.91 |
|  | Visit 3 | 7.10 | -3.00 | 10.10 | 178.31 |
|  | Visit 4 | 6.80 | 1.20 | 5.60 | 443.82 |
|  | Visit 5 | 2.20 | -4.20 | 6.40 | 495.29 |
| FFMI (kg^.^h^-2^) | Visit 2 | 1.00 | -0.40 | 1.40 | 797.45 |
|  | Visit 3 | 1.30 | -0.80 | 2.10 | 857.58 |
|  | Visit 4 | 2.00 | 0.30 | 1.70 | 1462.00 |
|  | Visit 5 | 1.60 | 0.30 | 1.30 | 2438.37 |
| Handgrip (kg) | Visit 2 | 4.10 | 5.20 | -1.10 | -1014.94 |
|  | Visit 3 | 4.50 | 3.60 | 0.90 | 2001.02 |
|  | Visit 4 | 5.20 | 4.10 | 1.10 | 2259.46 |
|  | Visit 5 | 10.40 | 2.70 | 7.70 | 411.67 |
| 6MWT (m) | Visit 2 | 95.00 | 13.00 | 82.00 | 13.62 |
|  | Visit 3 | 143.00 | 54.00 | 89.00 | 20.24 |
|  | Visit 4 | 170.00 | 17.00 | 153.00 | 16.24 |
|  | Visit 5 | 179.00 | 59.00 | 120.00 | 26.42 |
| sHPN: supplemental home parenteral nutrition, ICER: incremental cost-effectiveness ratio, Qol: quality of life, BMI: body mass index, FFM: fat free mass, FFMI: fat free mass index, 6MWT: six minutes walking test | | | | | |
